# Humanin ameliorates diabetes‐induced testicular damage in a streptozotocin‐induced mouse model

**DOI:** 10.1113/EP093912

**Published:** 2026-07-12

**Authors:** Munevver Gizem Hekim, Nalan Kaya Tektemur, Mete Ozcan

**Affiliations:** ^1^ Department of Physiology, Faculty of Medicine Firat University Elazig Turkey; ^2^ Department of Histology and Embryology, Faculty of Medicine Firat University Elazig Turkey; ^3^ Department of Biophysics, Faculty of Medicine Firat University Elazig Turkey

**Keywords:** diabetes mellitus, humanin, testicular damage

## Abstract

Diabetes mellitus is a major metabolic disorder closely associated with oxidative stress and male reproductive dysfunction. Humanin, a mitochondria‐derived peptide, has been reported to exert cytoprotective, anti‐apoptotic and antioxidant effects in various disease models; however, its role in diabetes‐induced testicular damage remains unclear. This study aimed to investigate the effects of humanin on oxidative stress parameters in seminal vesicle fluid and histopathological alterations in testicular tissue in a streptozotocin (STZ)‐induced diabetic mouse model. A total of 40 adult male Balb/C mice were randomly assigned into four groups: Control, Humanin (HN), STZ, and STZ+HN (*n* = 10 per group). Diabetes was induced via intraperitoneal administration of STZ (150 mg/kg). Humanin (4 mg/kg) was administered intraperitoneally for 15 days following diabetes induction. Total antioxidant status (TAS), total oxidant status (TOS), and glutathione (GSH) levels in seminal vesicle fluid were measured using ELISA. Testicular tissues were evaluated histopathologically and histomorphometrically. STZ‐induced diabetes resulted in a significant decrease in TAS and GSH levels and an increase in TOS levels in seminal vesicle fluid (*P *< 0.05). Humanin treatment significantly increased TAS and GSH levels while reducing TOS levels compared to the STZ group (*P *< 0.05). Histopathological analysis demonstrated seminiferous tubule degeneration, germ cell loss, interstitial oedema, and vascular congestion in diabetic mice, whereas these alterations and associated histomorphometric abnormalities were significantly ameliorated following humanin treatment. Humanin attenuated diabetes‐induced oxidative imbalance and testicular histopathological damage in STZ‐induced diabetic mice. These findings suggest that humanin may exert protective effects against diabetes‐associated testicular injury. Further studies are required to elucidate the underlying molecular mechanisms and validate these findings in broader experimental settings.

## Abstract (highlights)

- **What is the central question of this study?**
  Humanin is known for its ameliorative role in reducing mitochondrial dysfunction and oxidative stress in various disease models: does it have a similar protective role against diabetes‐induced testicular damage?
- **What is the main finding and its importance?**
  Humanin significantly restored oxidative balance in the seminal vesicle of diabetic mice by increasing TAS and GSH levels and reducing TOS levels, and markedly improved testicular histopathological damage. These findings suggest that humanin may be associated with protection against diabetes‐associated testicular injury.

## 1 INTRODUCTION

Diabetes and obesity are rapidly increasing metabolic disorders worldwide, affecting multiple organ systems (Yilmaz et al., 2025). Diabetes is characterized by hyperglycaemia, insulin resistance, and/or insulin deficiency, leading to long‐term complications across various organs (Sun et al., 2022). Diabetes affects many systems in the body, including the reproductive system. The male reproductive system is regulated by the hypothalamo‐pituitary neuroendocrine axis and may be affected by various hormones, drugs and factors (Khoubbieh et al., 2023; Sandal et al., 2015; Tan et al., 2026; Yilmaz et al., 2006). Diabetes negatively affects male fertility by causing testicular damage and a decrease in sperm quality and number (Alves et al., 2013; Pergialiotis et al., 2016).

Oxidative stress is well known to be associated with male infertility (Aydin et al., 2010). Diabetes‐induced hyperglycaemia is associated with oxidative stress in the male reproductive system and may contribute to apoptotic processes (Maresch et al., 2018; Ramalho‐Santos et al., 2008). In addition, hyperglycaemia reduces the mitochondrial antioxidant capacity in the testes and impairs testicular function (Maresch et al., 2018; Palmeira et al., 2001). Diabetes causes dysfunction in mitochondria, which play a role in the formation of reactive oxygen species (Rolo & Palmeira, 2006). Mitochondrial dysfunction negatively affects testicular physiology and has been associated with impaired male reproductive capacity (Nakada et al., 2006).

Humanin is the first identified member of the mitochondria‐derived peptides (MDPs). It was initially isolated and characterized by the Japanese researcher Shigeo Hashimoto and colleagues as a neuroprotective peptide associated with Alzheimer's disease (Hashimoto et al., 2001). Humanin has been reported to exert protective effects in various disease models, potentially through modulation of oxidative stress, mitochondrial function and inflammatory pathways (Abozaid et al., 2023; Cai et al., 2021; Charununtakorn et al., 2016; Coradduzza et al., 2023; Hazafa et al., 2021; Li et al., 2020). Given its established role in mitigating mitochondrial dysfunction and oxidative stress in different disease models, humanin may have a similar protective potential against diabetes‐related testicular damage (Hazafa et al., 2021; Kumfu et al., 2016; Qin et al., 2018). Beyond these cytoprotective properties, humanin has also been implicated in male reproductive biology. Humanin and its analogues have been reported to preserve spermatogenesis and attenuate germ‐cell apoptosis in experimental models of reproductive injury, while humanin expression in sperm has been associated with semen quality parameters, including sperm concentration and motility (Surampudi et al., 2015; Wang et al., 2024). Specifically, no studies have directly evaluated the effects of humanin on testicular damage in streptozotocin‐induced diabetic mouse models.

In this study, it was hypothesized that humanin, a mitochondria‐derived peptide with reported cytoprotective and antioxidant properties, would reduce oxidative stress parameters and ameliorate diabetes‐induced histopathological alterations in testicular tissue and seminal vesicle fluid. Furthermore, humanin administration was anticipated to help preserve testicular structure and oxidative balance in diabetic male mice.

## 2 METHODS

### 2.1 Ethical approval

This study protocol was approved by the Fırat University Local Ethics Committee for Animal Experiments (dated 9 October, 2019, protocol number 2019/47 and decision number 2019/19), and all experimental procedures were conducted in accordance with the principles of the *Guide for the Care and Use of Laboratory Animals* and the animal experiment policies of *Experimental Physiology*. Animals had ad libitum access to standard rodent chow and tap water and were housed under controlled environmental conditions (21 ± 1°C, 60 ± 5% humidity, 12 h light–dark cycle; lights on at 07.00 h).

### 2.2 Animals

In the experiments, 40 adult male Balb/C mice, aged 6–8 weeks and weighing 30 ± 5 g, were used. Sample size was determined using G*Power software based on an α level of 0.05 and statistical power of 80% to ensure adequate statistical power. To minimize selection bias, animals were allocated into experimental groups using a computer‐assisted simple randomization algorithm (MedCalc 12.7.0 for Windows) based on initial body weight. After a 7‐day acclimatization period, animals were allocated into four groups: Control, Humanin (HN) (4 mg/kg), Streptozotocin (STZ), and STZ + HN (4 mg/kg) (*n* = 10 per group). (Figure 1).

**FIGURE 1:**
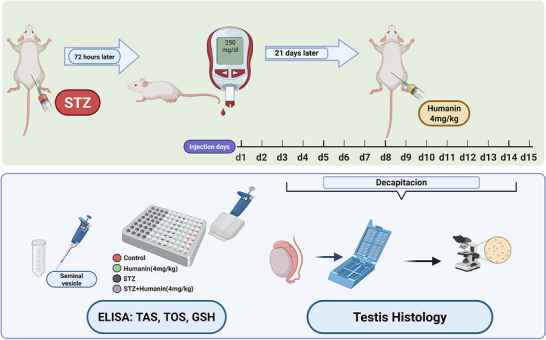
Flowchart of experimental design. Prepared using BioRender.com.

### 2.3 Streptozotocin‐induced diabetes model in mice

Streptozotocin (STZ) (Sigma Aldrich, St Louis, MO, USA, product no: S0130) was dissolved in 0.4 mL (0.1 M) sodium citrate buffer (pH 4.5). Normoglycaemic mice were injected intraperitoneally (i.p.) with STZ at a dose of 150 mg/kg. Seventy‐two hours later, blood glucose levels were measured using a manual glucometer (Optima, Hsinchu, Taiwan). Blood samples were collected from the tail vein of the mice, and all measurements were performed between 09.00 and 10.00 h. Mice with blood glucose levels exceeding 250 mg/dL were included in the study (Ozcan et al., 2022).

Following STZ administration, animals were monitored daily for body‐weight changes, general activity, grooming behaviour, and signs of distress, including persistent piloerection or reduced mobility. Mice exhibiting severe deterioration, including >20% body‐weight loss, markedly reduced activity or persistent piloerection, were predefined as meeting humane endpoint criteria and would have been excluded from the study. However, no animals met these criteria during the experimental period.

### 2.4 Humanin administration

HN administration was initiated 3 weeks after STZ administration. The HN used in the study (catalogue no: AS‐60886) was obtained from AnaSpec Inc. (Fremont, CA, USA). Prior to administration, HN was dissolved in phosphate buffered saline (PBS) (Sigma‐Aldrich, product no: P4417). The HN and STZ+HN groups received daily i.p. injections of HN for 15 days between 09.00 and 10.00 h. The humanin dose (4 mg/kg) and 15‐day administration protocol used in the present study were adopted from previous experimental studies reporting antioxidant and cytoprotective effects of humanin in metabolic and oxidative stress‐related conditions (Bilgin et al., 2025; Bulut et al., 2023). HN treatment was initiated 3 weeks after STZ administration, following establishment of the diabetic condition.

### 2.5 Histological evaluations and sample collection

After 15 days of humanin administration, animals were anaesthetized with ketamine (70 mg/kg) and xylazine (8 mg/kg) administered i.p., and following confirmation of adequate anaesthesia, euthanasia was performed by decapitation. Tissue collection was carried out immediately thereafter. The testes and seminal vesicles were carefully dissected. Following dissection, the testicular tissues fixed in Bouin's solution were washed through a gradually increasing ethanol series. Washed tissues were subjected to the routine histological follow‐up procedure and then embedded in paraffin (Sigma‐Aldrich Paraplast embedding medium). Sections of 5 µm thickness from the prepared paraffin blocks were mounted on ground glass slides. Slides were stained with haematoxylin–eosin (H&E). Stained sections were evaluated and photographed using the Leica imaging system (DM2500, Leica, Wetzlar, Germany).

Testicular damage was evaluated semi‐quantitatively in each section using a histopathological scoring system [0: no lesion (0%–25%), 1: very mild (26%–50%), 2: moderate (51%–75 %) and 3: severe lesion (76%–100%)]. Histological scoring was performed manually under light microscopy by a blinded investigator, without the use of automated image analysis software. In addition, histomorphometric analysis was performed by measuring seminiferous tubule diameter and germinal epithelium thickness in 200 randomly selected seminiferous tubules per group under ×20 magnification.

Following dissection, seminal vesicle contents were collected into Eppendorf tubes. The tissues were transferred into separate tubes containing 3 mL PBS and incubated for 10 min with gentle agitation at 2–3 min intervals. The seminal vesicles were subsequently removed, and the recovered fluids were combined and centrifuged at 2790 × g for 10 min. The resulting supernatants were stored at −80°C until biochemical analysis.

### 2.6 Biochemical analysis

Total antioxidant status (TAS) (E‐BC‐K801‐M, Elabscience, Houston, TX, USA), total oxidant status (TOS) (E‐BC‐K802‐M, Elabscience, Houston, TX, USA) and glutathione (GSH) (E‐EL‐M0026, Elabscience, Houston, TX, USA) levels in the seminal vesicle supernatants were measured using enzyme‐linked immunosorbent assay (ELISA). The concentrations of the samples were calculated by comparing their optical densities with a standard curve.

### 2.7 Statistical analysis

Statistical analyses were performed using GraphPad Prism version 10.5.0 (GraphPad Software, Boston, MA, USA). The normality of data distribution was assessed using the Shapiro–Wilk test. Homogeneity of variances was evaluated using the Brown–Forsythe test. Since the assumption of equal variances was not met, comparisons among groups were performed using Welch's one‐way ANOVA. Multiple comparisons were conducted using Dunnett's T3 *post hoc* test. Statistical reporting and figure presentation were revised to improve transparency and compliance with the journal's statistical guidelines. Statistical significance was predefined at an alpha level of 0.05, and exact statistical results, including ANOVA statistics and corresponding *P*‐values, were incorporated where appropriate. Graphical data were generated using GraphPad Prism software (version 10.5.0) and presented as violin plots to allow visualization of data distribution and variability across groups. Significant between‐group comparisons were indicated directly within the figures.

## 3 RESULTS

### 3.1 Physiological characterization of the diabetic model

STZ administration successfully induced diabetes, resulting in marked hyperglycaemia and body‐weight reduction compared with non‐diabetic groups (Table 1). No significant differences were observed between the control and HN groups. Although mice in the STZ+HN group remained hyperglycaemic, blood glucose levels were significantly lower than those observed in the STZ group.

**TABLE 1:** Physiological characterization of experimental groups.

Group	Initial body weight (g)	Final body weight (g)	Blood glucose (mg/dL)
Control	30.2 ± 4.2	35.1 ± 3.7^a^	85.6 ± 7.1^a^
Humanin (HN)	31.1 ± 3.9	34.4 ± 3.8^a^	89.5 ± 7.6^a^
STZ	30.4 ± 4.4	26.8 ± 4.0^b^	405.1 ± 21.4^b^
STZ+HN	31.8 ± 4.0	28.2 ± 4.5^b^	319.8 ± 24.5^c^

*Note*: Values are presented as means ± SD. Different superscript letters indicate statistically significant differences between groups (Welch's one‐way ANOVA followed by Dunnett's T3 *post hoc* test, *P* < 0.05).

### 3.2 Humanin improved oxidative stress markers

After 15 days of humanin administration, TAS, TOS and GSH levels were evaluated in seminal vesicle fluid samples obtained from the mice. TAS levels in the control, HN, STZ and STZ+HN groups were determined as 6.12 ± 0.94, 6.04 ± 0.87, 3.21 ± 0.62 and 5.74 ± 0.81 µmol/L, respectively. TAS levels were markedly decreased in the STZ group compared to the control group, whereas a significant increase was observed in the STZ+HN group following humanin administration. In addition, TAS levels in the HN‐only group were found to be comparable to those of the control group. The differences among the groups were statistically significant (*F*(3,36) = 32.62, *P* < 0.0001) (Figure 2).

**FIGURE 2:**
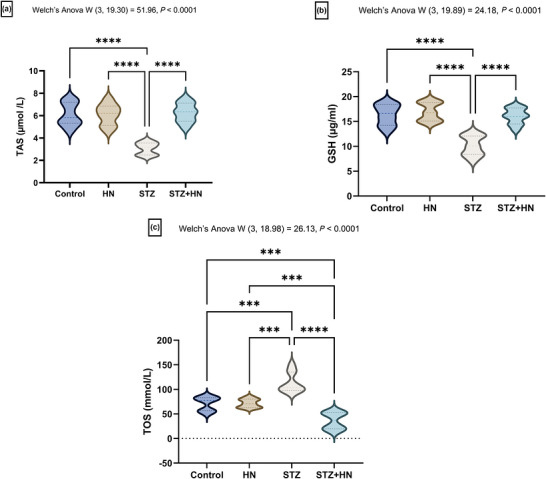
Effect of chronic humanin administration on antioxidant parameters in the seminal vesicle TAS levels (a), GSH levels (b) and TOS (c) levels (****P < 0.0001).

TOS levels in the control, HN, STZ and STZ+HN groups were found to be 69.84 ± 12.63, 71.25 ± 10.14, 112.47 ± 28.16 and 81.36 ± 17.94 mmol/L, respectively. TOS levels were markedly elevated in the STZ group compared to the control group, whereas a significant decrease was observed in the STZ+HN group following humanin treatment. Furthermore, TOS levels in the HN‐only group were similar to those of the control group. Statistical analysis revealed significant differences among the groups (*F*(3,36) = 40.12, *P* < 0.0001) (Figure 2).

GSH levels in the control, HN, STZ and STZ+HN groups were determined as 16.41 ± 1.97, 16.88 ± 1.75, 10.32 ± 1.88 and 15.28 ± 1.63 µg/L, respectively. GSH levels were markedly reduced in the STZ group compared to the control group, whereas Humanin administration significantly increased GSH levels in the STZ+HN group. In addition, GSH levels in the HN‐only group were comparable to those of the control group. Significant differences among the groups were observed for GSH levels (*F*(3,36) = 28.18, *P* < 0.0001) (Figure 2).

### 3.3 Humanin improved histological alterations

When the sections from the Control and HN groups were evaluated under light microscopy, the testicular tissues exhibited a normal histological appearance (Figure 3a,d). In the STZ+HN group, marked histological improvement with a near‐normal tissue appearance was observed (Figure 3e,f). In contrast, sections from the STZ group showed immature germ cells sloughed into the lumen of the seminiferous tubules, as well as interstitial oedema and vascular congestion (Figure 3b,c).

**FIGURE 3:**
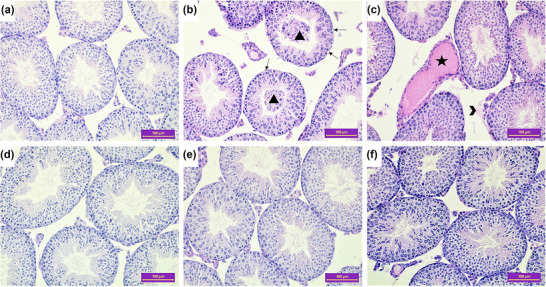
Histological appearance of testicular tissue (H&E). (a) Control group: normal appearance of germinal epithelium, Sertoli cells, basement membrane and interstitial area. (b) STZ group: immature germ cells sloughed into the lumen of seminiferous tubules (triangle). (c) STZ group: interstitial oedema (arrowhead) and vascular congestion in the interstitial area (asterisk). (d) HN group: normal appearance of germinal epithelium, Sertoli cells, basement membrane and interstitial area. (e, f) STZ+HN group: near normal appearance of germinal epithelium, Sertoli cells, basement membrane and interstitial area.

Histomorphometric evaluation demonstrated that STZ‐induced diabetes caused significant deterioration in seminiferous tubule morphology. Seminiferous tubule diameter values in the control, HN, STZ and STZ+HN groups were determined as 231.1 ± 19.82, 235.5 ± 19.88, 199.6 ± 15.77 and 260.8 ± 22.18 µm, respectively. A marked decrease in seminiferous tubule diameter was observed in the STZ group compared to the control group, whereas a significant increase was detected in the STZ+HN group following humanin administration. In addition, seminiferous tubule diameter values in the HN‐only group were found to be comparable to those of the control group. Statistical analysis revealed significant differences among the groups (*P* < 0.0001).

Germinal epithelium thickness values in the control, HN, STZ and STZ+HN groups were found to be 67.67 ± 7.59, 69.26 ± 7.449, 51.53 ± 4.881 and 85.34 ± 1.406 µm, respectively. Germinal epithelium thickness was markedly reduced in the STZ group compared to the control group, whereas humanin administration significantly increased germinal epithelium thickness in the STZ+HN group. Furthermore, germinal epithelium thickness values in the HN‐only group were similar to those observed in the control group. Significant differences among the groups were observed with respect to germinal epithelium thickness (*P* < 0.0001) (Figure 4).

**FIGURE 4:**
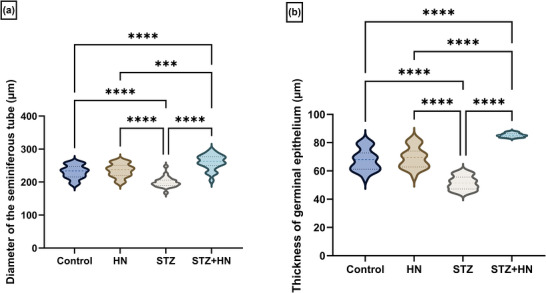
Effect of chronic humanin administration on testicular histopathology. (a) Diameter of the seminiferous tube in testis. (b) Thickness of germinal epithelium in testis (****P < 0.0001).

## 4 DISCUSSION

This study demonstrated that diabetes mellitus induces testicular structural and oxidative alterations in male mice, and that humanin administration attenuated seminiferous tubule degeneration, interstitial oedema and vascular congestion while improving oxidative stress parameters.

Type 1 diabetes mellitus is well known to be a major source of oxidative stress, along with other metabolic disorders. The primary reason for this is that hyperglycaemia activates abnormal metabolic pathways that lead to excessive production of reactive oxygen species (ROS) in many tissues, including the testes (Venditti et al., 2024). Under conditions of increased oxidative stress, ROS induce cellular damage through mechanisms such as lipid peroxidation, protein damage and DNA damage. Mitochondria play a critical role in this process, and oxidative stress ultimately leads to mitochondrial dysfunction (Cameron et al., 1985; Guneli et al., 2008). Previous studies have reported reductions in oxidative stress markers and inflammatory responses following humanin treatment in neuropathic and metabolic disease models (Bilgin et al., 2025; El Kattawy et al., 2022; Hazafa et al., 2021; Kelestemur et al., 2024; Kumfu et al., 2016; Qin et al., 2018). In the present study, humanin treatment produced significant improvements in oxidative stress parameters in the seminal vesicle fluid of diabetic mice. The observation that TAS and GSH levels approached control values, together with reduced TOS levels, suggests antioxidant potential associated with humanin treatment. Although oxidative stress markers were evaluated in seminal vesicle fluid rather than directly in testicular tissue, these measurements were intended to provide an indirect assessment of reproductive tract oxidative status and should be interpreted within this context. Collectively, these findings suggest that humanin may contribute to protection against diabetes‐associated oxidative and histopathological alterations. Although humanin‐treated diabetic mice remained hyperglycaemic, the partial reduction in blood glucose levels observed in the STZ+HN group raises the possibility that systemic metabolic improvement may have contributed, at least in part, to the local findings.

It is well known that diabetes leads to both morphological and functional impairments in testicular structures. In STZ‐induced diabetes models, significant degeneration of seminiferous tubules, loss of germ cells, increased apoptosis, and a marked reduction in testicular weight have been demonstrated (Guneli et al., 2008; Luo et al., 2022). These morphological alterations are considered indicative of impaired spermatogenesis (Kus et al., 2000, 2005).

Humanin is abundantly expressed in the testes of various species, including rats, mice and humans (Lei and Rao, 2022). Humanin and its analogues have been reported to ameliorate chemotherapy‐ and stress‐related reproductive injury, preserve spermatogenesis and attenuate germ‐cell apoptosis in experimental models of male reproductive damage (Surampudi et al., 2015; Wang et al., 2024). Furthermore, in vitro studies have shown that humanin supplementation may improve post‐thaw sperm motility and functional parameters (Gemeda et al., 2026; Pande et al. 2022; Yang et al., 2019). These observations support the biological relevance of humanin within the male reproductive system. Humanin may also exert anti‐inflammatory and anti‐apoptotic effects in various disease models (Bulut et al., 2023; El Kattawy et al., 2022; Hazafa et al., 2021; Kelestemur et al., 2024; Kumfu et al., 2016); however, these pathways were not directly assessed in the present study and should therefore be considered speculative in the context of diabetes‐induced testicular injury. These findings may be interpreted in light of previously proposed mechanisms associated with humanin activity. These observations may provide context for the histological improvements observed in our study. The marked germ cell loss and structural deterioration observed in the STZ group were largely ameliorated in the humanin‐treated groups, suggesting a protective role of humanin.

This study has several limitations. A major limitation is the absence of functional reproductive assessments, including sperm parameters, fertility outcomes, testicular weight and hormonal measurements. Therefore, the present findings should be interpreted as evidence of structural and biochemical protection rather than direct preservation of reproductive function. Furthermore, the underlying molecular mechanisms responsible for the protective effects of humanin were not directly investigated. In particular, inflammatory, apoptotic, and mitochondrial pathways were not experimentally assessed and therefore remain speculative in the context of the present study. Circulating or tissue humanin concentrations were also not measured; therefore, direct confirmation of systemic or local humanin exposure was beyond the scope of the present study. Future studies are warranted to further elucidate these mechanisms and validate the present findings in broader experimental settings.

In conclusion, this study demonstrated that humanin significantly attenuated diabetes‐induced testicular damage in an STZ‐induced diabetic mouse model. Humanin administration restored oxidative balance in the seminal vesicle by increasing TAS and GSH levels while decreasing TOS levels, and concomitantly improved histopathological alterations in testicular tissue. These findings suggest that humanin treatment was associated with protection against diabetes associated testicular injury. However, further studies are required to elucidate the underlying molecular mechanisms and confirm these findings in broader experimental models.

## AUTHOR CONTRIBUTIONS

Munevver Gizem Hekim: conceptualization, methodology, writing—review and editing. Nalan Kaya Tektemur: methodology, writing – review and editing. Mete Ozcan: conceptualization, methodology, supervision, project administration, funding acquisition, writing – review and editing. All authors approved the final version of the manuscript; agree to be accountable for all aspects of the work in ensuring that questions related to the accuracy or integrity of any part of the work are appropriately investigated and resolved; and all persons designated as authors qualify for authorship, and all those who qualify for authorship are listed.

## CONFLICT OF INTEREST

The authors declare that they have no known competing financial interests or personal relationships that could have appeared to influence the work reported in this paper.

## GENERATIVE AI STATEMENT

Generative artificial intelligence (AI) tools were used solely for language editing, grammar checking, and improvement of manuscript readability. Specifically, ChatGPT (OpenAI, GPT‐5 series) was used to assist with language refinement and editorial suggestions. All scientific content, study design, data analysis, interpretation of results and final manuscript decisions were performed and verified by the authors, who take full responsibility for the content of the manuscript.

## Data Availability

Data will be made available on request.
